# Vγ9Vδ2 T-cell immunotherapy in blood cancers: ready for prime time?

**DOI:** 10.3389/fimmu.2023.1167443

**Published:** 2023-04-18

**Authors:** Claudia Giannotta, Federica Autino, Massimo Massaia

**Affiliations:** ^1^Laboratorio di Immunologia dei Tumori del Sangue (LITS), Centro Interdipartimentale di Biotecnologie Molecolari “Guido Tarone”, Dipartimento di Biotecnologie Molecolari e Scienze per la Salute, Università Degli Studi di Torino, Torino, Italy; ^2^Struttura Complessa (SC) Ematologia, Azienda Ospedaliera (AO) S. Croce e Carle, Cuneo, Italy

**Keywords:** Vγ9Vδ2 T cells, immunotherapy, adoptive cell transfer, unconventional T cells, blood cancers

## Abstract

In the last years, the tumor microenvironment (TME) has emerged as a promising target for therapeutic interventions in cancer. Cancer cells are highly dependent on the TME to growth and evade the immune system. Three major cell subpopulations are facing each other in the TME: cancer cells, immune suppressor cells, and immune effector cells. These interactions are influenced by the tumor stroma which is composed of extracellular matrix, bystander cells, cytokines, and soluble factors. The TME can be very different depending on the tissue where cancer arises as in solid tumors *vs* blood cancers. Several studies have shown correlations between the clinical outcome and specific patterns of TME immune cell infiltration. In the recent years, a growing body of evidence suggests that unconventional T cells like natural killer T (NKT) cells, mucosal-associated invariant T (MAIT) cells, and γδ T cells are key players in the protumor or antitumor TME commitment in solid tumors and blood cancers. In this review, we will focus on γδ T cells, especially Vγ9Vδ2 T cells, to discuss their peculiarities, pros, and cons as potential targets of therapeutic interventions in blood cancers.

## Introduction

γδ T cells are equipped with a T-cell Receptor (TCR) composed of a γ-chain (TRG) and a δ-chain (TRD). The genes encoding TRG and TRD undergo somatic DNA recombination of variable (V), diversity (D, only in TRD) and joining (J) elements during γδ T cell maturation in the thymus (1). γδ TCR and αβ TCR are structurally similar and associated with the same subunits of the CD3 complex which, however, are arranged differently and characterized by unique glycosylation patterns and other minor peculiarities (2, 3). One major difference are the antigens recognized by αβ and γδ T cells and the modality of antigen recognition which is not dependent on the major histocompatibility complex (MHC) in γδ T cells (2, 4). This feature is particularly exciting from the perspective of using γδ T cells as a source for adoptive cell transfer (ACT) or chimeric antigen receptor (CAR)-T cells because MHC-independency reduces the risk of graft-versus-host disease (GvHD) and helps the development of “off-the shelf” cellular products (5).

In humans, γδ T cells represent 1-5% of blood circulating cells (6). Their development begins early during gestation (5-7 weeks), initially in the liver, and after 8 weeks of gestation also in the thymus (7). Later on, γδ T cells colonize predetermined mucosal and epithelial locations to contribute to tissue homeostasis and immune responses against pathogens (8).

Human γδ T cells can be divided in three main subsets: Vδ1^+^ cells, Vδ2^+^ cells and Vδ3^+^ cells (2). Vδ2^+^ T cells are the predominant γδ T-cell population in the PB of adult humans (9, 10). They are characterized by the expression of the semi-invariant Vγ9Vδ2 TCR made up of a public germline CDR3γ sequence and a more diverse CDR3δ sequence (11–14). Vδ1^+^ and Vδ3^+^ cells are commonly found in mucosal epithelial tissues, and in the liver, even if small amounts can also be detected in PB (2). *In vitro*, CD8^+^ Vδ1^+^ T cells which can recognize tumor-associate antigens in an MHC-dependent manner have been generated from human cord blood hematopoietic stem/progenitor cells (HSPC) using the OP9-DL system (15). The importance of γδ T cells in the clearance of pathogens (8, 16, 17) and cancer immunosurveillance (18–20) is very well acknowledged. However, γδ T cells can also negatively affect the outcome of immune responses to pathogens and tumor cells depending on the tissue microenvironment that they have colonized, the cytokines and soluble factors they are exposed to, and the multifaceted interactions engaged with bystander cells and the extracellular matrix (21–23). This functional plasticity can lead to the acquisition of regulatory functions in the tumor microenvironment (TME) leading to immune suppression and protumor functions. Accumulation of CD39^+^ γδ T cells has been reported in colorectal cancer (23), and interleukin (IL)-17 producing Vδ1^+^ T cells have been identified as major promoters of tumor progression and metastatization in humans (24–26). Regulatory γδ T cells have also been reported in blood cancers and associated with poor overall survival (27, 28). Fewer data are available about Vγ9Vδ2 T cells and other Vδ2^-^ cells (29). Recently, we have reported that bone marrow (BM) Vγ9Vδ2 T cells in multiple myeloma (MM) patients are dysfunctional, but they do not exert suppressor functions and do not produce IL-17 (30), whereas Lo Presti et al. have reported IL-17 producing Vγ9Vδ2 T cells in the TME of patients with squamous cell carcinoma (31).

In this review, we will discuss the peculiarities and vulnerabilities of Vγ9Vδ2 T cells to behave as antitumor immune effector cells, and the pros and cons to build autologous or allogenic immune-based interventions on these cells.

## Activation and functional characteristics of Vγ9Vδ2 T cells

Vγ9Vδ2 T cells can recognize supraphysiological concentrations of phosphoantigens (pAgs) produced by pathogens or eukaryotic cells *via* the mevalonate pathway (Mev) or Mev-independent pathways of isoprenoid biosynthesis (32). The Mev-independent pathways (MEP/DOXP or Rohmer pathway) are restricted to eubacteria, cyanobacteria, plants, and apicomplexan protozoa (33). The prototype pAg generated in the Mev pathway is isopentenyl pyrophosphate (IPP). IPP is over-produced by stressed cells and cancer cells and promotes the selective activation of Vγ9Vδ2 T cells (34). The mechanisms of pAgs recognition by Vγ9Vδ2 T cells are very different from the canonical MHC-antigen complex recognition by αβ T cells and not yet fully resolved. Three immunoglobulin superfamily members, butyrophilin 3A1 (BTN3A1), butyrophilin 3A2 (BTN3A2), and butyrophilin 2A1 (BTN2A1) are involved in pAgs presentation and Vγ9Vδ2 T-cell activation (8, 35–38). The intracellular 30.2 domain of BTN3A1 senses pAg accumulation in antigen presenting cells (APCs) or target cells (8, 36) and promotes an inside-out modification of the extracellular domains. Once modified, BTN3A1 is stabilized by BTN3A2 and binds to the Vδ2 and γ-chain TCR regions of Vγ9Vδ2 T cells. At the same time, BTN2A1 provides a costimulatory signal *via* interactions with BTN3A1 and the germline-encoded regions of the Vγ9 chain on the opposite TCR side (37–39).

BTN3A1 and BTN2A1 are also expressed on the cell surface of Vγ9Vδ2 T cells. This implies that Vγ9Vδ2 T cells can self-activate each other without the intervention of APCs or target cells if there are sufficient pAgs in the extracellular space that can be internalized by the ATP-binding cassette transporter A1 (ABCA1). Self-activation is associated with CD107a upregulation and increased interferon-γ (IFNγ) production, potentially leading to Vγ9Vδ2 T-cell fratricide (40, 41). This undesired side-effect can partially explain why Vγ9Vδ2 T-cell based immune interventions have fallen short of expectations in the clinical setting (41).

Aminobisphosphonates (NBP) like zoledronic acid (ZA), and alkylamines enhance the ability of APCs and cancer cells to activate Vγ9Vδ2 T cells by increasing the intracellular production and extracellular release of IPP *via* inhibition of the farnesyl diphosphate synthase in the Mev pathway (42–45). Vγ9Vδ2 cells can also be activated by natural killer (NK) receptors like the natural killer 2D receptor (NKG2D) and the DNA X accessory molecule 1 (DNAM-1). The former interacts with MICA, MICB, and ULBP1-4, while the latter interacts with Nectin-2 and PVR. These interactions contribute to the induction of cytotoxic responses and cytokine production (25). Vγ9Vδ2 T cells can also express NKp44 which is involved in cytotoxicity against myeloma cells lacking NKG2D ligands (46, 47). Other NK receptors, such as NKp30, NKp40 and NKp46 can also contribute to the antitumor functions of Vδ1 and Vδ2 T cells (32). Upon activation, Vγ9Vδ2 T cells can exert a wide range of functions typical of both adaptive and natural immunity, including cytolytic functions, chemokines and cytokines production. In addition, they can behave as cellular adjuvants to support antigen-specific immune responses mediated by B cells and MHC-restricted αβ T cells (2, 45, 48–52).

Vγ9Vδ2 T cells can also exert regulatory functions to terminate immune reactions and prevent autoimmunity *via* IL-10 production and the immune checkpoint (ICP) - immune checkpoint ligands (ICP-L) axes (43, 53).

Based on their maturation status, four distinct subsets of Vγ9Vδ2 T cells have been identified after pAgs stimulation (43). Naïve CD45RA^+^CD27^+^ Vγ9Vδ2 T cells produce low amount of IFNγ, and they can differentiate into CD45RA^-^CD27^+^ central memory (CM) Vγ9Vδ2 T cells with higher proliferation capacity after pAgs stimulation. CM cells can further differentiate into CD45RA^-^CD27^-^ effector memory (EM) cells that produce high levels of IFNγ and tumor necrosis factor-α (TNFα) (54). EM cells or, alternatively, CM cells in the presence of IL-15, can differentiate into late effector memory CD45RA^+^CD27^-^ T cells (TEMRA) characterized by high cytotoxic activity, low proliferative capacity, and modest IFNγ production (43, 54). TEMRA cells can be further divided in two subsets based on CD45RA expression levels: CD27^-^CD45RA^hi^ and CD27^-^CD45RA^int^ cells. The former are reminiscent of functionally exhausted cells, while the latter are the “classical” TEMRA cells mentioned above (55). The maturation process of Vγ9Vδ2 T cells is highly influenced by the microenvironment in which they are resident and the stimuli they are exposed to. In the presence of tumor cells, the maturation pathway can be redirected to immune senescence and/or functional exhaustion which are tumor permissive conditions (30).

## Vγ9Vδ2 T cells in cancer: A delicate balance between antitumor and protumor functions

The antitumor activity of Vγ9Vδ2 T cells encompasses: 1) direct killing of cancer cells through granzyme B (GzmB) and perforin (Prf) secretion; 2) antibody-dependent cellular cytotoxicity (ADCC) dependent on CD16 expression; 3) Fas/FasL-mediated cell death; 4) production of cytokines like IFNγ and TNFα; 5) interactions with other TME-resident immune cells (25, 48, 56, 57). Vγ9Vδ2 T cells, can cross-present tumor antigens to αβ CD8^+^ T cells to boost antigen-specific IFNγ production and increase antitumor T-cell response (58). Vγ9Vδ2 T cells can also upregulate MHC and co-stimulatory molecules after *in vitro* IPP stimulation. This APCs-like phenotype allows Vγ9Vδ2 T cells to prime CD4^+^ T cells, shifting their polarization towards a Th1 antitumor profile (49). We and others have shown that Vγ9Vδ2 T cells can deliver co-stimulatory signals to dendritic cells (DCs) after *in vitro* ZA stimulation that increase the frequency of antigen-specific CD8^+^ αβ T cells and concurrently restrain the expansion of IL-2-dependent regulatory T cells (Tregs). Altogether, these data indicate that Vγ9Vδ2 T cells can behave as cellular adjuvants to rally a wide range of immune reactions against cancer cells (52, 59, 60) mediated by innate and adaptive immune effector cells, including B cells, neutrophils, and NK cells (57). Vγ9Vδ2 T cells can provide B-cell help to promote antibody production and immunoglobulin class switching (57, 61). IL-21 in combination with (E)-4-Hydroxy-3-methyl-but-2-enyl pyrophosphate (HMB-PP) can induce a T_FH_-like Vγ9Vδ2 T-cell differentiation leading to increased IgM and IgG production by B cells (61). Soluble factors released by activated Vγ9Vδ2 T cells trigger neutrophil migration, phagocytic ability and α-defensin release which can exert antitumor activity in the TME (62). IPP-activated Vγ9Vδ2 T cells upregulate CD137L that can engage CD137 on the surface of NK cells and enhance the cytotoxic antitumor activity against squamous cell carcinoma of head and neck and lymphoma cell lines (63).

Despite this wide array of direct and indirect antitumor properties, Vγ9Vδ2 T cells are very early targeted and neutralized by cancer cells, especially in the TME. In MM, BM Vγ9Vδ2 T cells are PD-1^+^ TIM-3^+^, and anergic to pAgs stimulation (30, 64). These dysfunctions are long-lasting and already detectable in monoclonal gammopathy of undetermined significance (MGUS) (64). PD-1^+^ BM MM Vγ9Vδ2 T cells combine phenotypic, functional, and TCR-associated alterations consistent with chronic exhaustion and immune senescence, not easily reversible by single or even by dual ICP blockade (30). Interestingly, ICP^+^ Vγ9Vδ2 T cells maintain the ability to produce IFNγ and to secrete GzmB and Prf in MM, acute myeloid leukemia (AML), and other cancers (30, 65, 66). It is unclear whether these cells are still able to provide some kind of immune surveillance in the TME, but the partial retention of immune effector functions suggests that their immunocompetence is not irreversibly lost, and hopefully recoverable by appropriate manipulation.

The functional plasticity of Vγ9Vδ2 T cells implies a constant risk of switching from antitumor to protumor function (25, 48). Depending on the cytokines they are exposed after activation, Vγ9Vδ2 T cells can polarize into Th1-like, Th2-like, Th17-like, T_FH_-like, Treg-like, T_APCS_-like phenotypes (43, 67–69). The input to undertake one way of differentiation rather than another is also influenced by the tissue environment, including cancer cells. Similarly to what has been reported on total γδ T cells in breast, colon, and pancreatic cancer (21, 26, 56, 70), Th17-like Vγ9Vδ2 T cells with protumor functions have been identified in the TME and associated with a negative outcome in squamous cell carcinoma (31). In the presence of IL-21, Vγ9Vδ2 T cells can become CD73^+^ and suppress the antitumor activity of conventional T cells *via* the adenosine suppressive circuitry (67). PD-L1 upregulation in the presence of IPP and IL-15 is another potent immune suppressor mechanism operated by Vγ9Vδ2 T cells against αβ T cells (71, 72). CD86 can also be used by Vγ9Vδ2 T cells to suppress αβ T cells *via* CTLA-4 and restrain their antitumor activity (72).

Vγ9Vδ2 T cells, in turn, can become easy targets of immune suppressor cells like myeloid-derived suppressors cells (MDSC) or bone marrow stromal cells (BMSC) that are often increased in the TME and are PD-L1^+^, as we have recently shown in MM (64, 73). The supraphysiological IPP production and release by BMSC *via* ABCA-1 can also contribute to the functional exhaustion of Vγ9Vδ2 T cells in the TME of MM (30, 40).

**Figure 1:**
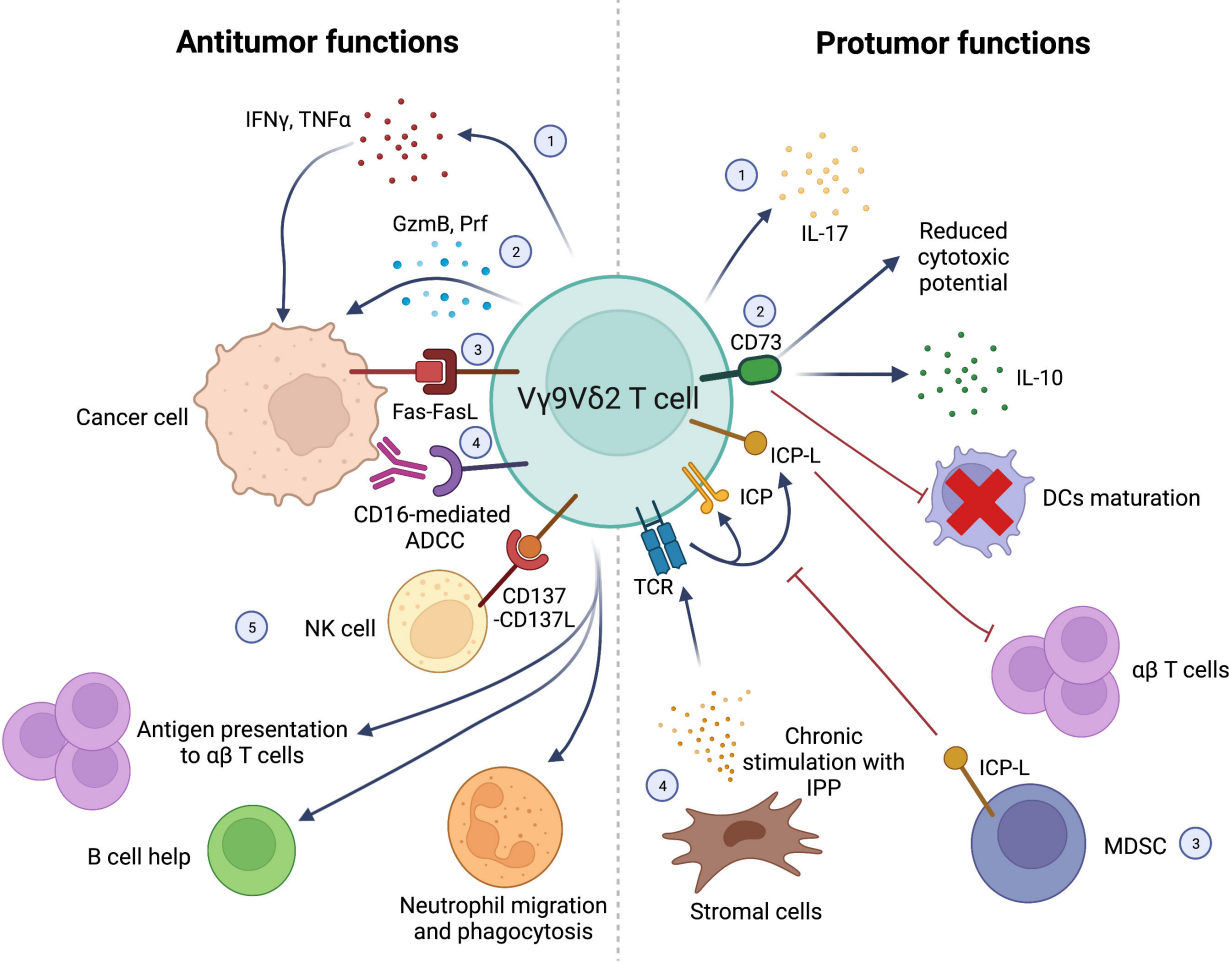
Schematic representation of antitumor (left) and protumor (right) functions of Vγ9Vδ2 T cells. *Antitumor functions:* 1) IFNγ and TNFα production; 2) direct killing of cancer cells *via* GzmB and Prf production; 3) cancer cell killing *via* Fas-FasL interactions; 4) CD16-mediated ADCC; 5) synergistic interactions with other immune cells in the TME: NK cells stimulation *via* CD137L expression; Ag presentation to αβ T cells; B cell help; stimulation of neutrophils’ migration, phagocytosis and α-defensin release. *Protumor functions:* 1) IL-17 production; 2) CD73 expression leading to IL-10 production, decreased Vγ9Vδ2 T-cell cytotoxic activity and impaired DCs maturation; 3) Negative regulation of Vγ9Vδ2 by T-cells MDSC expressing ICP-L (i.e. PD-L1); 4) Chronic stimulation of Vγ9Vδ2 TCR with IPP produced by stromal cells leading to exhaustion and suppression of αβ T cells’ function through ICP/ICP-L axis. Interferon γ (IFNγ), Tumor Necrosis Factor α (TNFα), Granzyme B (GzmB), Perforin (Prf), Antibody-dependent cell cytotoxicity (ADCC), Antigen (Ag), Interleukin-17 (IL-17), Interleukin-10 (IL-10), Dendritic cells (DCs), Myeloid-derived suppressor cells (MDSC), Immune Checkpont-Ligands (ICP-L), Programmed Death-Ligand 1 (PD-L1), Isopentenyl pyrophosphate (IPP), Immune Checkpoint (ICP). Created with BioRender.com.

Antitumor and protumor functions of Vγ9Vδ2 T cells are represented in **Figure 1**.

Interestingly, blood cancer cells are more susceptible to the antitumor activity of Vγ9Vδ2 T cells than solid tumors (34, 56). Possible mechanisms are the enhanced Mev pathway activity and the increased expression of stress-induced self-ligands (34). Another major role is played by the TME which is very different in solid and blood cancer. The emergence of protumor Vγ9Vδ2 T cells has more often been reported in the former, whereas in the latter Vγ9Vδ2 T cells are mainly dysfunctional and chronically exhausted, but not fully differentiated into Vγ9Vδ2 T cells with protumor functions (30, 74–76).

## Vγ9Vδ2 T cells as candidates for immunotherapy: A failed promise or inappropriate engagement?

The unique properties of Vγ9Vδ2 T cells have raised a great interest as potential candidates for immune-based interventions in solid tumors and blood cancers. Vγ9Vδ2 T-cell activation can be induced by a wide array of ligands making possible to target cancer cells devoid of specific tumor-associated antigens (TAA) or tumors with a limited mutational burden. Moreover, a broad antitumor reactivity could prevent the emergence of tumor variants leading to immune escape and tumor relapse (77).

MHC independency is another major feature making Vγ9Vδ2 T cells safer effector cells than αβ T cells in the context of allogenic hematopoietic stem-cell transplantation (allo-HCT) or other mismatched adoptive immunotherapy approaches. Vγ9Vδ2 T cells can exert effective graft-*versus*-tumor (GvT) activity with minimal GvHD activity which still is a major cause of early and late morbidity and mortality after allo-HCT (78). MHC-independent recognition of TAA should also limit the ability of cancer cells to evade immune recognition *via* MHC down-regulation (79).

The frequency of Vγ9Vδ2 T cells in the PB is low, but still significantly higher than any other MHC-restricted TAA-specific αβ T cells, and pAgs stimulation is a polyclonal stimulation recruiting all Vγ9Vδ2 T cells and not only selected clonal or subclonal populations. MHC-independency gives the possibility to develop off-the-shelf cell products from healthy donors bypassing both the time-consuming and expensive manufacturing of personalized cell products, and the immune dysfunctions affecting Vγ9Vδ2 T cells from cancer patients. Allogeneic and haploidentical Vγ9Vδ2 T cells have already been used in solid tumors and hematological malignancies without major adverse effects (80–84). Burnham et al. have shown that Vγ9Vδ2 T cells from multiple donors can be mixed and stimulated with ZA and IL-2 after αβ T-cell depletion without inducing fratricide or affecting their expansion and functional activation (85). Multidonor preparations could circumvent the risk to produce inadequate numbers of activated Vγ9Vδ2 T cells from healthy donors who are poor responders to pAg stimulation (approximately 5-10%). However, safety of multidonor infusions has not been tested in the immunotherapy setting, with the exception of cord blood cells, and the risk of uncontrolled alloreactivity remains a major concern (85). Lastly, pAgs-activated Vγ9Vδ2 T cells have been shown *in vitro* to behave as cellular adjuvants with the ability to engage immune effector cells of adaptive immunity and boost their antitumor responses (52, 57, 58, 60).

Despite these excellent premises, Vγ9Vδ2 T-cell based immune interventions have not hit the target. Early approaches have used NPB like pamidronate and ZA to induce Vγ9Vδ2 T-cell activation *in vivo* followed by IL-2 to support proliferation and expansion. Synthetic pAgs like bromohydrin pyrophosphate (BrHPP) and 2-methyl-3-butenyl-1-pyrophosphate (2M3B1PP) have been produced to increase the affinity for Vγ9Vδ2 T cells and extend their half-life after *in vivo* injection. Synthetic pAgs have been associated *in vivo* with monoclonal antibodies (mAbs) like rituximab, alemtuzumab, and obinutuzumab to boost ADCC in B-cell malignancies, based on the *in vitro* findings that pAgs-activated Vγ9Vδ2 T cells upregulate FcγR expression (86–88).

Early approaches of adoptive immunotherapy have also relied on the combination of pAgs and IL-2 to induce the *ex-vivo* activation of autologous Vγ9Vδ2 T cells. This approach has been tested in MM showing minimal toxicity, but unsatisfactory clinical results (89). The adjuvant properties of Vγ9Vδ2 T cells and their capacity to promote the activation of tumor-specific MHC-restricted αβ T cells has been investigated in a small number of elderly AML patients. These patients have been treated with DCs co-pulsed with WT1 peptide and ZA with some evidences of clinical benefit (90–92).

In conclusion, Vγ9Vδ2 T-cell based immunotherapy has proven safe and well tolerated in blood cancers, but unable to achieve deep and long-lasting responses (32, 93, 94). Failing clinical expectations has stimulated further research to understand the mechanisms exploited by tumor cells to escape Vγ9Vδ2 T-cell recognition and killing, especially in the TME (95, 96), and which strategies are worth investigating to empower their antitumor activity.

A critical point is the immune fitness of Vγ9Vδ2 T cells in cancer patients. We and others have shown that about 50% of PB Vγ9Vδ2 T cells from Chronic Lymphocytic Leukemia (CLL), MM, and other blood cancer patients are unable to respond to pAgs stimulation (97, 98). Naïve/CM/EM/TEMRA subset redistribution, ICP upregulation, immune senescence, and functional exhaustion due to chronic stimulation are some of the mechanisms responsible for Vγ9Vδ2 T-cell dysfunctions (30, 64). Unique to Vγ9Vδ2 T cells is the chronic stimulation operated by the supra-physiological IPP concentrations that are released in the TME by BMSC, and to a lower extent by myeloma cells (40). At the same time, the supra-physiological IPP concentrations can license the suppressor activity of Vγ9Vδ2 T cells restraining the antitumor activity of conventional αβ T cells *via* the PD-1/PD-L1 axis (71).

Interestingly, we have shown that Vγ9Vδ2 T-cell dysfunctions in the TME of MM patients are highly persistent and not reverted even in the remission phase when myeloma cells have disappeared (64). One reason is that the TME remains strongly committed to immune suppression as shown by the persistence of high numbers of PD-L1^+^ MDSC, PD-L1^+^ BMSC, and PD-L1^+^ endothelial cells (EC). Moreover, the disease status strongly influences the reactivity of BM MM Vγ9Vδ2 T cells to pAgs stimulation and the response to ICP blockade. At diagnosis, the combination of PD-1 and TIM-3 blockade allows a partial recovery of Vγ9Vδ2 T-cell immune effector functions; in the remission phase, single PD-1 blockade is moderately effective, whereas PD-1 and LAG-3 blockade is the only combination to be minimally effective in relapsed MM (30).

These data indicate that TME-resident Vγ9Vδ2 T cells are probably not the better targets for cell-based immune interventions in the absence of appropriate *ex-vivo* or *in vivo* manipulation correcting their dysfunctions. This is an interesting difference with tumor-infiltrating lymphocytes (TIL) which have been deemed to be very well-suitable for cellular immunotherapy. The assumption is that, at least in solid tumors, tumor-reactive clones have already been primed in the TME and they can be recruited more effectively against cancer cells (99). Moreover, frequency of TIL is much higher than that of γδ T cells in the TME facilitating their selective isolation and expansion (100).

A possible alternative to TME-resident Vγ9Vδ2 T cells is the *in vivo* or *ex-vivo* recruitment of circulating Vγ9Vδ2 T cells. Side-by-side comparison of PB and BM Vγ9Vδ2 T cells in MM patients has shown that the former are functionally preserved slightly better than the latter. We and others have shown that approximately 50% of MM and CLL patients retain PB Vγ9Vδ2 T cells that can be stimulated by pAgs (97, 98). Interestingly, in the others the anergy can be reverted with ZA-stimulated DCs that provide huge quantities of IPP and costimulatory signals (45, 97, 101). In CLL, pretreatment of PB Vγ9Vδ2 T cells with ibrutinib promotes a Th1 differentiation with enhanced antitumor activity, probably mediated by ITK inhibition as previously reported in conventional αβ T cells (101).

The use of PB Vγ9Vδ2 T cells is not devoid of drawbacks. One is the progressive decline in the capacity to respond to reiterated ZA stimulations as shown in MM patients after autologous stem cell transplantation (102), and pediatric acute leukemia patients receiving haploidentical αβ T-cell depleted stem cell transplantation (103). Another critical aspect is the inadvertent expansion of CD4^+^ T cells with a regulatory phenotype, as shown in neuroblastoma patients treated with ZA+IL-2 to intentionally activate Vγ9Vδ2 T cells *in vivo* (104).

Vγ9Vδ2 T-cell MHC independency gives the possibility to use allogeneic cells from the PB of healthy donors (105). Haploidentical γδ T cells have been infused in 4 patients with refractory hematological malignancies followed by *in vivo* stimulation with ZA and IL-2. None of the patients suffered from acute or chronic GvHD providing the proof in principle that allogeneic Vγ9Vδ2 T cells can safely be transferred and stimulated *in vivo* without inducing any undesired alloreactivity (82). These preliminary data have been validated in a large series of patients with advanced stage liver and lung cancer patients who received allogeneic Vγ9Vδ2 T cells without any significant adverse effects (e.g., immune rejection, cytokine storm, or GvHD effects) (81).

Although very exciting, also the use of Vγ9Vδ2 T cells from healthy donors is not exempt from disadvantages and pitfalls. One is the unexpected induction of immune suppressive activity against conventional αβ T cells after repeated pAgs stimulation (71). Another pitfalls are the unpredictable consequences of transferring Vγ9Vδ2 T cells which have been forced to respond to pAgs *via* noncanonical stimulation. For example, IL-21 has been reported to promote the expansion of Vγ9Vδ2 T cells from non-responder donors after ZA stimulation (85). Unfortunately, IL-21 can also induce Vγ9Vδ2 T cells with immune suppressive and protumor functions exerted *via* the CD73/adenosine-dependent circuit (67).

Altogether, these data indicate that both TME-resident and PB Vγ9Vδ2 T cells are very sensitive to stimuli delivered by TME, cytokines, and pAgs. Their functional plasticity is a great plus, but at the same time a great risk to inadvertently induce an undesired protumor activity if not properly managed (30, 106).

## Strategies to bring Vγ9Vδ2 T-cell immune interventions to prime time

Over the last few years, we have seen an enormous acceleration in the knowledge of immune escape mechanisms together with great advances in the design of therapeutic mAbs, and the development of genetically engineered immune effector cells. These very exciting progresses are revolutionizing cancer immunotherapy including Vδ1 and Vγ9Vδ2 T-cell based approaches (94, 107).

Several approaches are under preclinical or clinical investigation to rescue the immune fitness of Vγ9Vδ2 T cells in cancer patients. Anti-ICP/ICP-L mAbs have been used *in vitro* to improve pAgs reactivity and immune effector functions of TME-resident Vγ9Vδ2 T cells in MM (30, 64), AML (65) and follicular lymphoma (FL) (108). The agonistic humanized anti-BTN3A mAb ICT01 is under investigation in advanced-stage solid tumors and hematological malignancies (109). Bispecific T-cell engagers antibodies (BiTe) are also under investigation to redirect cytotoxic Vγ9Vδ2 T-cell activity against cancer cells. The bispecific Vγ9/CD123 antibody has been shown to recruit and redirect Vγ9Vδ2 T cells against autologous AML blasts *in vitro* and in a xenograft mouse model (110). Similar results have been reproduced *in vitro* and in a xenograft mouse model with the bispecific Vγ9Vδ2/CD40 antibody in CLL and MM patients (111). CD1d is another tumor-associated antigen which can be targeted in CLL with a CD1d-specific Vγ9Vδ2-T cell engager made by single-domain antibodies (VHH). Interestingly, this bispecific VHH does not affect pAg reactivity giving the possibility to boost the antitumor activity of Vγ9Vδ2 T cells with ZA (112). Van Diest et al. have developed a bispecific molecule which exploits the natural predisposition of Vγ9Vδ2 T cells to recognize cancer cells by linking the extracellular domains of tumor reactive Vγ9Vδ2 TCR to a CD3-binding moiety. This bispecific molecule confers to conventional αβ T cells the capacity to recognize cancer cells *via* pAgs without the limitations imposed by MHC restriction and/or MHC downregulation (113).

A great effort is also ongoing to optimize the use of Vγ9Vδ2 T cells from healthy donors. In this case, strategies are dedicated to improve the efficacy of *in vitro* expansion protocols and to reinforce the capacity of Vγ9Vδ2 T cells to survive *in vivo* and to exert a prolonged antitumor activity. One area of research is focused on the discovery of novel NBP and synergistic interactions with other compounds. Tetrakis-pivaloyloxymethyl 2-(thiazole-2-ylamino) ethylidene-1,1-bisphosphonate (PTA) is a novel bisphosphonate prodrug which activates Vγ9Vδ2 T cells more efficiently than ZA (114), while vitamin C and its derivatives can enhance the activation and differentiation of human Vγ9Vδ2 T cells (115). A wise and careful selection of cytokines is also critical to promote the expansion of antitumor Vγ9Vδ2 T cells, and not the undesired expansion of Vγ9Vδ2 T cells with protumor or immune suppressor functions (85, 116).

The use of feeder cells is another workable tool to improve the efficacy of *in vitro* Vγ9Vδ2 T-cell expansion protocols (117–120). Side-by-side comparison of ZA + IL-2 *versus* K562-based artificial antigen-presenting cells (aAPCs) has shown in mouse models that the latter induces Vγ9Vδ2 T cells with stronger antitumor activity and enhanced capacity to survive *in vivo* (118). However, the superiority of aAPCs is challenged by the risk to induce an excessive IL-17A release leading to the differentiation of protumor Vγ9Vδ2 T cells (118, 121). Costimulation with ZA + IL-2 in addition to aAPCs can overcome this undesired bias and support the expansion of large numbers of memory Vγ9Vδ2T cells with low ICP expression that are prone to persist *in vivo* after infusion (119). This approach has been improved by introducing an intermediate step to remove αβ T cells in between the first stimulation with ZA + IL-2 and the second one with aAPCs and ZA + IL-2. This strategy allows the manufacturing and expansion from healthy donors of huge numbers of highly pure Vγ9Vδ2 T cells (117). The cytotoxic activity of adoptively transferred Vγ9Vδ2 T cells can be strengthened with mAbs to relieve ICP/ICP-L-dependent immune suppression (122, 123), and/or with agonistic anti-BTN3A 20.1 mAb or BiTes to boost antitumor immune effector functions (124).

Alternative strategies to potentiate antitumor effector functions of Vγ9Vδ2 T cells take advantage of their ability to recognize stress-induced self-ligands *via* killer activating receptors (KAR) like NKG2D. This ability is counterbalanced by the expression of killer inhibitory receptors (KIR) (34), highlighting the importance to develop strategies that upregulate KAR and/or downregulate KIR in Vγ9Vδ2 T cells. Attempts to tilt the balance in favor of KAR range from nanobiomaterial-based strategy to conventional drugs. Lin et al. have shown *in vitro* that chitosan nanoparticles enhance Vγ9Vδ2 T-cell antitumor functions by upregulating NKG2D, CD56, FasL, and Prf secretion (125). Upregulation of NKG2D-ligands (NKG2D-L) in cancer cells can be a complementary strategy. Conventional drugs like temozolomide, doxorubicin, and 5-fluorouracyl can sensitize cancer cells from solid tumors to Vγ9Vδ2 T cells by inducing the upregulation of Fas, TRAIL-R1, and TRAL-R2 that are recognized by Vγ9Vδ2 T cells *via* NKG2D and TRAIL (126, 127). These results have been reproduced with bortezomib in AML and acute T-cell lymphoblastic leukemia. Story et al. have shown that bortezomib enhances the recognition and killing of leukemia cells by *ex-vivo* activated Vγ9Vδ2 T cells from healthy donors by increasing NKG2D/NKG2D-L interactions (128). Unfortunately, these drugs can also be toxic to Vγ9Vδ2 T cells. The easiest way to skip this inconvenience is to give chemotherapy before Vγ9Vδ2 T-cell activation *in vivo* or before infusion of *ex-vivo* activated Vγ9Vδ2 T cells (127). A more cumbersome approach is to genetically engineer Vγ9Vδ2 T cells to confer resistance to cytotoxic drug (126). The extracellular release of NKG2D-L is another mechanism exploited by cancer cells to elude NKG2D-dependent immune surveillance, especially after exposure to cytotoxic drugs. Prevention of NKG2D-L shedding is another strategy that can be used to improve the efficacy of combinations with cytotoxic drugs (129).

The immune adjuvant properties of Vγ9Vδ2 T cells are also of renewed interest. Early studies have focused on their ability to boost MHC-restricted antitumor immune responses mediated by conventional CD8^+^ T cells (92). More recently, tumor cell/Vγ9Vδ2 T-cell fusions have been developed to mimic tumor cell/DC fusions already tested in MM and AML (130–132). In this approach, DCs are replaced by pAg-activated Vγ9Vδ2 T cells to combine their abilities to support adaptive immune responses and to exert antitumor activity, a plus compared with DCs which lack any direct antitumor activity. Wang et al. have validated this approach *in vitro* by generating osteosarcoma/Vγ9Vδ2 T-cell fusions that induce cytokines production and support antitumor immune responses mediated by conventional αβ T cells (133).

Sharing innate-like and adaptive-like immune functions makes Vγ9Vδ2 T cells very attractive candidates for genetic engineering (134). Vγ9Vδ2 T cells have successfully been armed with CAR to target the B-cell Maturation Antigen (BCMA) in MM and CD123 in AML (135, 136). Interestingly, *in vitro* data and *in vivo* mouse models have shown that, unlike conventional anti-CD19 CAR-T cells, ZA-stimulated anti-CD19 Vγ9Vδ2 CAR-T cells from healthy donors can target both CD19^+^ and CD19^-^ allogeneic leukemia cells *via* the non-specific MHC-independent cytotoxic activity elicited by pAgs stimulation (137). It is worth investigating whether the retained ability to target CD19^-^ leukemic cells can be exploited to prevent the disease relapse observed in patients treated with conventional anti-CD19 CAR-T cells. In addition, CAR-transduced Vδ2 T cells do not lose their property to behave as professional APCs and to cross-present processed peptides to αβ T cells (138).

ZA-stimulated Vγ9Vδ2 T cells are also excellent candidates for subsequent RNA-transfection with tumor-specific TCRs or CARs (139). Likewise, αβ T cells can be engineered to express γδ TCRs with high capacity to sense BTN3A1 and other conformational changes induced by intracellular pAgs accumulation in tumor cells (140). γδ TCR chains are very strong competitors of αβ TCR chains for the assembly of the TCR/CD3 complex (141) preventing the formation of αβ/γδ heterodimers and limiting the expression of endogenous αβ TCRs (142). The availability of GMP-grade anti-αβ TCR beads gives the possibility to deplete non- and poorly-engineered T cells yielding to a population of untouched engineered immune cells with high purity and substantially reduced “off-target” effects (143, 144). These T cells engineered to express a defined γδ T cell receptor (TEGs) have been shown to limit leukemic cell growth *in vitro* (140) and to recognize and kill myeloma cells in a 3D model (145). In addition, CD4^+^ Vγ9Vδ2 TCR-transduced αβ T cells retained the ability to induce DC maturation (140). The high affinity γ9δ2TCR clone 5 has demonstrated to be effective against AML blasts in PD-X models (146) and has been selected within the TEG format as a clinical candidate (TEG001) for a phase I clinical trial in patients with relapsed and refractory AML and MM (NTR https://www.trialregister.nl/trial/6357).

A side-by-side comparison of conventional αβ T cells and Vγ9Vδ2 T cells transduced with TCRs or CARs to target melanoma cells has shown similar antigen-specific cytotoxic activity, but the latter retain also their intrinsic ability to lyse MHC-deficient cells. Moreover, the cytokines pattern released by transduced Vγ9Vδ2 T cells predicts a lower risk of cytokine release syndrome and autoimmunity compared with transduced αβ T cells (139). Lastly, Vγ9Vδ2 T cells have been transfected with NKT cell-derived TCR to create bi-potential innate lymphocytes combining NKT and Vγ9Vδ2 effector functions including cytotoxicity against glycolipid-expressing target cells and K562 cells (147). Saura-Esteller et al. and Mensurado et al. have recently reviewed the clinical studies exploiting BiTes and engineered Vγ9Vδ2 T cells in cancer immunotherapy (94, 148).

Vγ9Vδ2 T-cell-based immunotherapy, like any other immunotherapy, can benefit from interventions shaping the TME to meet the metabolic requirements of immune effector cells at the expense of immune suppressor cells and cancer cells. In mouse cancer models, Lopes et al. have shown that protumor (IL-17^+^) and antitumor (IFNγ) γδ T cells are characterized by distinct metabolic profiles: the former require mitochondrial metabolism, whereas the latter are almost exclusively glycolytic. As a consequence, antitumor activity of IFNγ^+^ γδ T cells can be boosted by glucose, whereas protumor activity of IL-17^+^ γδ T cells can be reinforced or weakened by regulating lipid metabolism (149). Indoleamine 2,3-dioxygenase 1 (IDO1) inhibition is another metabolic approach promoting Vγ9Vδ2 T-cell cytotoxicity against human breast cancer cells and pancreatic ductal adenocarcinoma (PDAC) cells by enhancing perforin production (150), degranulation, and cytokine production (151). The cytotoxic activity promoted by IDO inhibition can be further enhanced with bispecific antibodies targeting Vγ9Vδ2 T cells and PDAC cells (151).

Hypoxia is a metabolic TME alteration compromising the cytotoxic activity Vγ9Vδ2 T cells and promoting IL-17 production, and CD8^+^ T-cell inhibition *via* the PD-1/PD-L1 axis (152). In brain tumors, it has been shown that metformin alleviates tumor hypoxia and reinvigorates the antitumor function of γδ T cells by inducing NKG2D upregulation (20). Arginase I inhibition is another metabolic approach that can indirectly promote the antitumor activity of Vγ9Vδ2 T cells by restraining the suppressor activity of MDSC (73, 153). We have recently reviewed the role of metabolic checkpoints compromising the immune competence of Vγ9Vδ2 T cells in MM, and the possible interventions to recover their antitumor activity (154).

Vγ9Vδ2 T cell-based immunotherapy can also be enhanced by increasing tumor sensitivity and immunogenicity. Chemotherapeutic compounds (i.e. doxorubicin and oxaliplatin), proteasome inhibitors and immunomodulatory drugs (IMiDs) can induce immunogenic cell death (ICD) triggering adaptive immune responses through a set of danger signals (155). Combinatorial approaches with ICD-inducers can facilitate Vγ9Vδ2 T-cell recruitment and cytotoxic activity (127, 156). Since accelerated Mev-pathway affects the translocation on the cell surface of Calreticulin (CRT), an hallmark of ICD, NBP-mediated interruption of Mev-pathway could be also an effective strategy to promote the sensitivity of cancer cells to ICD (157).

**Figure 2:**
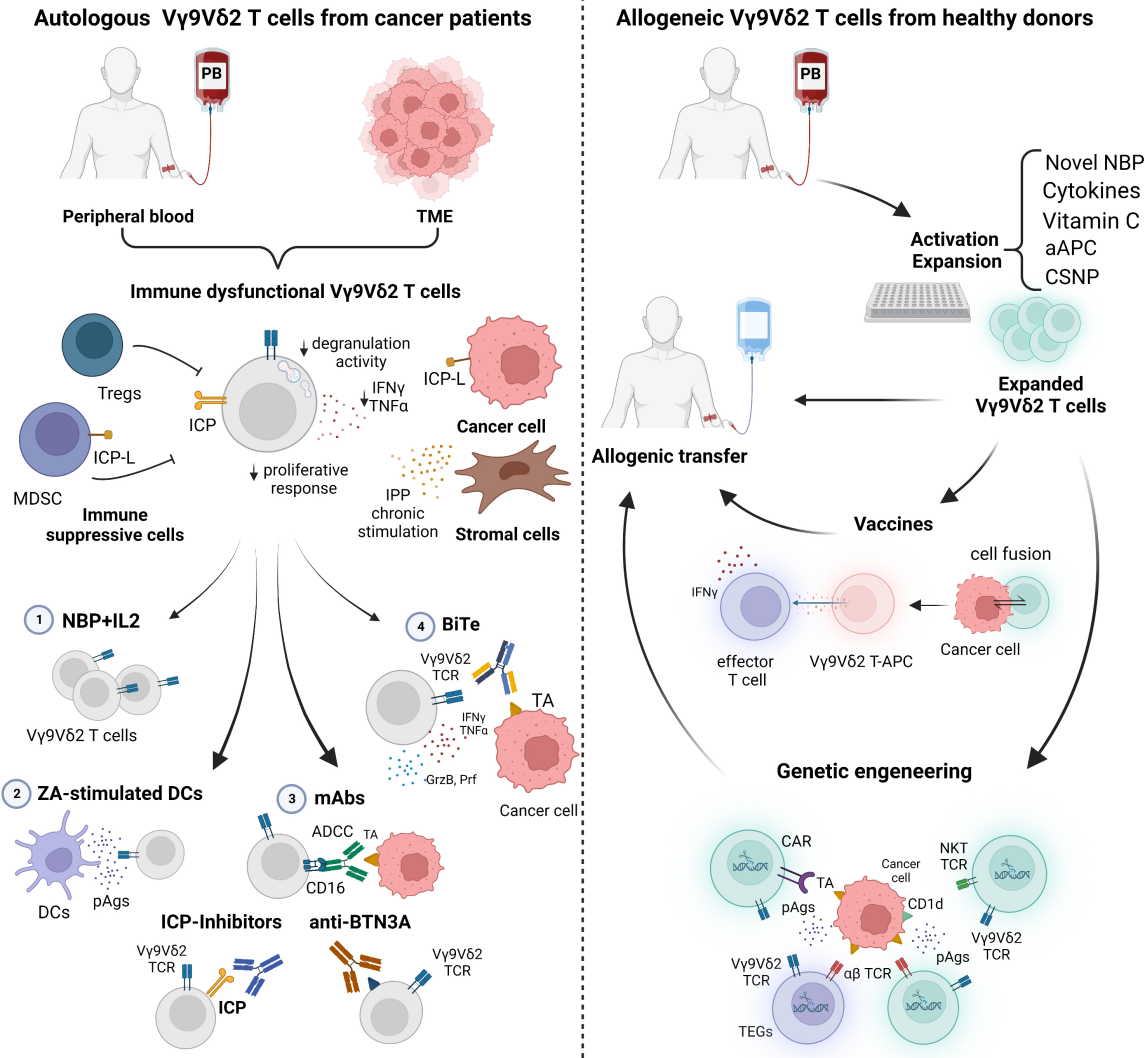
Current strategies to manipulate autologous and allogeneic Vγ9Vδ2 T cells for immunotherapy. *Left panel*: The immune fitness of patient-derived Vγ9Vδ2 T cells is compromised. Tumor microenvironment (TME)-resident and peripheral blood (PB) Vγ9Vδ2 T cells are characterized by immune checkpoint (ICP) expression, low proliferative response, decreased cytokine production (IFNγ and TNFα), and degranulation activity. Conventional approaches to rescue Vγ9Vδ2 T cells with 1) NBP+IL2 administration can be implemented with 2) ZA-stimulated dendritic cells (DCs) to enhance the amount of phosphoantigens (pAgs) locally available; 3) monoclonal antibodies (mAbs) to boost ADCC, to block ICP/ICP-L interactions, or to target BTN3A; 4) bispecific antibodies (BiTes). *Right panel*: Vγ9Vδ2 T cells from PB of healthy donors (Ctrl) can be manipulated *in vitro* for allogenic use. Novel NBP, selected cytokines, vitamin C, artificial antigen presenting cells (aAPCs) or chitosan nanoparticles (CSNP) can be used to improve Vγ9Vδ2 T-cell expansion and activation. Expanded Vγ9Vδ2 T cells can be used for vaccination or genetic engineering. Created with BioRender.com.

**Figure 2** summarizes the *in vivo* and *ex-vivo* strategies currently under investigation to recover and fully exploit the antitumor activity of autologous and/or allogeneic Vγ9Vδ2 T cells.

## Conclusions

In conclusion, Vγ9Vδ2 T cells are very attractive candidates for cell-based immunotherapy in blood cancers. However, Vγ9Vδ2 T cells are also very sensitive to the TME and very easily reprogrammable to exert protumor functions or to undergo functional exhaustion and/or immune senescence. To fully exploit their unique antitumor properties, it is mandatory to protect Vγ9Vδ2 T cells from the pernicious influence operated by the TME and to fully recover their immune competence status.

## Author contributions

CG, FA and MM contributed to the writing of the manuscript, CG and FA designed the figures, MM revised the manuscript. All authors contributed to the article and approved the submitted version.
